# Strains of *Mycobacterium tuberculosis* differ in affinity for human osteoblasts and alveolar cells in vitro

**DOI:** 10.1186/s40064-016-1819-z

**Published:** 2016-02-24

**Authors:** Shrabanti Sarkar, Muyalo G. Dlamini, Debapriya Bhattacharya, Olubisi T. Ashiru, A. Willem Sturm, Prashini Moodley

**Affiliations:** Department of Infection Prevention and Control, Nelson R Mandela School of Medicine, School of Laboratory Medicine and Medical Science, College of Health Science, University of KwaZulu-Natal, 719 Umbilo Road, Durban, 4075 South Africa

**Keywords:** *Mycobacterium tuberculosis*, Extra-pulmonary tuberculosis, XDR F15/LAM4/KZN, Human osteoblasts (SaOS-2), Human alveolar epithelial cells (A549)

## Abstract

Although the lung is the primary site of infection of tuberculosis, *Mycobacterium tuberculosis* is capable of causing infection at other sites. In 5–10 % such extra-pulmonary tuberculosis is located in bone tissue of the spine. It is unknown whether host or microbial factors are responsible for the site where extra-pulmonary tuberculosis manifests itself. One MDR isolate belonging to strain F28, one susceptible F11 and one isolate each of susceptible, MDR and XDR F15/LAM4/KZN were cultured in Middlebrook 7H9 media. Human osteoblasts (SaOS-2) and human alveolar epithelial cells (A549) were exposed to these different isolates of *M. tuberculosis* and invasion capacity and intra-cellular multiplication rates were established. Mouse macrophage (MHS) cells exposed to *M. tuberculosis* H37Rv served as control. The invasion capacity of F15/LAM4/KZN representatives increased with the level of resistance. The F28 MDR strain showed similar invasion capacity as the XDR F15/LAM4/KZN for pulmonary epthelial cells, whilst the fully susceptible F11 strain displayed a propensity for osteoblasts. The differences observed may in part explain why certain strains are able to cause infection at specific extra-pulmonary sites. We postulated that the development of extra-pulmonary tuberculosis depends on the ability of the microbe to pass effectively through the alveolar epithelial lining and its affinity for cells other than those in pulmonary tissue.

## Background

Tuberculosis (TB) is one of the most important causes of morbidity and mortality in the world (Supply et al. 2001). Although drug susceptible TB is a curable disease, it is considered a global public health problem in developing countries (Dye et al. 1999). Worldwide, approximately nine million new TB cases and 1.6 million deaths occur annually (WHO 2012; Adlakha et al. 2012). *Mycobacterium tuberculosis* is the causative agent of TB. It is a facultative intracellular bacteria that begins the disease in the lung after inhalation of bacilli (Dannenberg 1982). Once infection is established, dissemination of the bacilli from the lung into the lymphatics or blood occurs resulting in spread of the infection to other part of the lungs and also to other organ systems (Dannenberg 1982). Macrophages are thought to be the principal host cell (Geiser 2002) which carry the bacilli from the alveolar space into interstitium and bloodstream (Bermudez et al. 2002). However, since there is a predominance of epithelial cells in the lung (Crandall and Kim 1991) these cells may be the first barrier that the organism faces (Adlakha et al. 2012). Earlier studies reported that *M. tuberculosis* adheres, invades and replicates within pulmonary epithelial cells (Ashiru et al. 2010; Bermudez and Goodman 1996; Mehta et al. 1996). While the lung is the primary site of infection, *M. tuberculosis* can establish infection in virtually any other organ of the body, known as extra-pulmonary tuberculosis. Patients co-infected with HIV show a four-fold higher risk of extra-pulmonary tuberculosis (Kruijshaar and Abubakar 2009). Globally extra-pulmonary tuberculosis accounts for 1–5 % of all TB cases but in South Africa 15 % of all recent cases of TB were extrapulmonary (WHO 2012; Trecarichi et al. 2012).

Osteoblasts originate from the mesenchymal stem cell (Stein et al. 1990; Gandhi et al. 2013). They play a major role in synthesis of bone matrix and regulate the activity of osteoclasts (Gay et al. 2000). It has been reported that *Staphylococcus aureus* and *M. tuberculosis*, which are common etiological agents of osteoarticular infections, can infect human osteoblasts in vitro (Jevon et al. 1999; Wright and Friedland 2002). Spinal tuberculosis (Pott’s disease) is one of the most devastating, destructive and frequently encountered extrapulmonary forms of the disease. It is characterized by chronic inflammation with massive bone destruction of the spinal vertebrae, causing fracture and collapse of the vertebrae, ultimately resulting in compression of the spinal column with risk of paralysis and neurological deficits (Hoshino et al. 2014).

It is unknown what role host and microbial factors play in the development of Pott’s disease and how this compares to pulmonary infection. Approximately 20 % of patients with pulmonary TB develop extra pulmonary infections and 60–75 % of patients with spinal TB have active or latent pulmonary tuberculosis (Garg and Somvanshi 2011). According to Herath et al., the globally reported prevalence of extra-pulmonary tuberculosis ranges from 17 to 52 %, with concurrent pulmonary involvement in up to 14 % (Herath and Lewis 2014).

The F15/LAM4/KZN genotype, first reported in the province of KwaZulu-Natal in the early 1990s, has developed into a family of closely related strains accompanied by progression from drug susceptible and multidrug-resistant (MDR) to extensively drug-resistant (XDR) (Pillay and Sturm 2007). This genotype has been associated with the largest outbreak of XDR TB reported globally (Gandhi et al. 2013; Pillay and Sturm 2007). The rapid spread of this strain with concomitant high mortality among HIV-infected patients in the Tugela Ferry region of KwaZulu-Natal province (Gandhi et al. 2006) suggests increased virulence. Other successful *M. tuberculosis* strain families are F11 and F28, which were found in more than 70 % of cases of drug-resistant TB in the rural regions of Boland-Overberg and Southern Cape-Karoo of the Western Cape Province in South Africa (Streicher et al. 2004). F11 is present in 21.4 % of all infected patients in the Western Cape communities, and is therefore at least as successful as the Beijing genotype which is responsible for 16.5 % of cases (Streicher et al. 2004; Victor et al. 2004). Among the patients with TB from the urban communities in Cape Town, the F28 strain causes disease in 9.7 % of cases. This strain family has not been reported from other parts of South Africa or other regions of Africa. Although F28 appears to be common in the Western Cape Province, comparison of the IS6110-RFLP and the international RIVM spoligotype database suggests that this family may be more wide spread globally than previously thought.

To determine whether F15/LAM4/KZN, F28 and F11 families of *M. tuberculosis* have a specific affinity for bone cells, we studied invasion and intracellular multiplication of pulmonary isolates of these strains in human osteoblasts (SaOS-2) and compared this with invasion and multiplication in pulmonary epithelial cells (A549) and alveolar macrophages (MHS). We also addressed the question whether affinity for osteoblasts in F15/LAM4/KZN strains increased in parallel with development of resistance by including susceptible, MDR, and XDR representatives.

## Methods

### *Mycobacterium tuberculosis* isolates and growth condition

The *M. tuberculosis* isolates used in this study were retrieved from the culture collection in the Department of Infection Prevention and Control, Nelson R Mandela School of Medicine, School of Laboratory Medicine and Medical Science, College of Health Sciences, University of KwaZulu-Natal. Three of these belonged to the F15/LAM4/KZN (KZN) family of which one was fully susceptible, one MDR and one XDR. One susceptible isolate belonged to the F11 and one MDR isolate to the F28 family (see Table 1). Laboratory strain H37Rv (ATCC 27294) was included for reference purposes. The isolates were cultured for approximately 3 weeks at 37 °C in a shaking incubator in Middlebrook 7H9 broth, supplemented with 10 % oleic acid, albumin, dextrose, catalase (OADC), 0.05 % Tween 80 and 0.5 % glycerol.

**Table 1:** Description of different clinical isolates of *Mycobacterium tuberculosis* used in the experiments (susceptible; MDR: multi-drug resistant; XDR: extensively drug resistant)

*Mycobacterium tuberculosis* family	Strain number	Resistant to
F15/LAM4/KZN	V4027	_
F15/LAM4/KZN	MODS 688	I, R, E, P
F15/LAM4/KZN	MODS 388	I, R, E, P, S, Eth, A, C, K, O
F28	TF 44949	I, R, E, P
LAM3/F11	TF 832	_

*I* isoniazid, *R* rifampin, *E* ethambutol, *P* pyrazinamide, *S* streptomycin, *Eth* ethionamide, *A* amikacin, *C* capreomycin, *K* kanamycin, *O* ofloxacin

### Inocula preparation

Cultures were harvested and single cell suspensions were obtained as previously described (Ashiru et al. 2010). Briefly, vortex agitated suspensions were allowed to stand at room temperature for 15 min. Thereafter, the top 6 ml was forced 10 times up and down through a 26 gauge needle and then filtered using a 5 µm Millipore filter. To determine the number of colony forming units (CFU) per ml, volumes of 10 µl of suspensions with optical density at 600 nm (OD 600) of 1 were plated in triplicate on OADC enriched Middlebrook 7H11 agar media (Difco).

### Cell lines

Three different cell lines were used for the experiments. A549 human type II alveolar epithelial cells (ATCCCCL185) were cultured in EMEM medium containing 25 mM of HEPES, 10 % heat-inactivated fetal bovine serum (FBS), 2 mM of l-glutamine; MH-S murine alveolar macrophage cells (ATCCCRL2019) in RPMI-1640 medium containing 25 mM of HEPES, 10 % FBS, 2 mM of l-glutamine and SaOS-2 human osteoblast cells (ATCC HTB-85) were grown McCoy’s 5a medium with 15 % FBS. Semi-confluent monolayers were detached by trypsinization using trypsine-versene (Cambrex BioScience). Trypan blue exclusion was used to determine the number of viable cells using a haemocytometer and 1 ml of the cell suspensions containing 1 × 10^5^ viable cells/ml were seeded into each well of a 24-well tissue culture plate. Experiments were done with A549 and MHS cells incubated for ±48 h at 37 °C in 5 % CO_2_ atmosphere at >90 % confluency, whereas SaOS-2 cells were incubated similarly for 72 h.

### Invasion and multiplication assay

Bacterial invasion was studied as described before (Ashiru et al. 2010) with modifications. Cells were washed thrice with phosphate buffered saline (PBS pH 7.3; Oxoid) maintained at room temperature, after which 1 ml cell-specific culture medium (maintained at room temperature) was added to each well. Following inoculation of the cells with the *M. tuberculosis* suspension at a multiplicity of infection (MOI) of 9-12 per cell, plates were incubated for 2 h at 37 °C.

To kill extracellular bacteria, aminoglycoside protection assays were performed. This involved exposing the inoculated cells to media containing amikacin (200 µg/ml) for 1 h. This concentration of amikacin was used after susceptibility screening had been carried out. Briefly, standardized *M. tuberculosis* inoculum was prepared in volumes of 1 ml as mentioned above using isolates with different susceptibility profiles (susceptible, MDR and XDR) and H37Rv. Each suspension was centrifuged at 3000×*g* for 20 min. The supernatants were discarded and replaced with 1 ml of media containing different concentrations of amikacin (20, 30, 40 and 50 µg/ml). This was followed by brief vortex agitation to resuspend the bacilli and thereafter, the bacterial suspensions were incubated for 1 h at 37 °C and plated onto OADC enriched Middlebrook 7H11 agar plates in triplicate and incubated for 3 weeks to determine the CFU per ml.

After the aminoglycoside protection assay, the cells were washed thrice with PBS, pH 7.3 to remove the amikacin and then lysed with 1 ml of 0.1 % Triton X-100 in distilled water for 20 min. The spent culture medium was plated on OADC enriched Middlebrook 7H11 agarplates and incubated for 3 weeks to check whether all extracellular bacteria were killed. Serial ten-fold dilutions of the lysate were prepared in Middlebrook 7H9 broth with 0.05 % Tween −80 and plated on OADC enriched Middlebrook 7H11 agar medium. The plates were incubated for 3 weeks in sealed CO_2_ permeable plastic bags at 37 °C and the percentage of bacterial invasion was determined based on the obtained colony counts.

To measure intra-cellular multiplication, separate wells with amikacin treated inoculated cells were further incubated in their specific growth media at 37 °C in 5 % CO_2_ atmosphere for different time periods. After specific time points (24, 48 and 72 h), the overlaying growth medium was removed and the infected cell monolayers were washed with PBS and lysed as described above. Ten-fold serial dilutions of the lysates were plated in triplicate on OADC enriched 7H11 plates and incubated for 3 weeks for viable count analysis.

### Statistical analysis

All data were analysed by one-way ANOVA (Post Hoc Test: Tukey HSD by means of SPSS software) or student t test. In all cases significance was accepted at *P* < *0.05*.

### Ethics approval

The study was approved by the Biomedical Research Ethics Committee of the University of KwaZulu-Natal (Reference no: BCA274/09).

## Results

### Invasion of *Mycobacterium tuberculosis* of osteoblasts and alveolar cell lines

No growth was observed following exposure of *M. tuberculosis* isolates to 30, 40 and 50 µg/ml of amikacin respectively including the XDR isolates. There was also no growth from the discarded media following the amikacin protection assay. This further confirmed that only internalized bacilli were reported. The amikacin concentration of 200 µg/ml used in this aminoglycoside protection assay is 100 times higher than the recommended breakpoint for resistance and 10 times higher than the reported concentration that killed extracellular bacilli (Ashiru et al. 2010) and this also kills XDR isolates (WHO 2009).

**Fig. 1:**
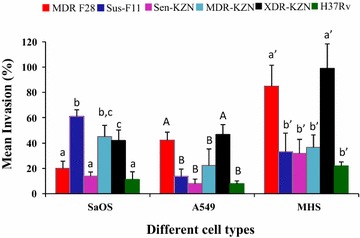
Comparison of the invasion capacity of different strains of *Mycobacterium* *tuberculosis* within human osteoblasts (SaOS-2) cells, epithelial cells (A549) and murine alveolar macrophages (MHS) at MOI 9-12. After 2 h of infection cells were incubated with antibiotics to kill extracellular bacteria. Cells were lysed and plated on 7H11 agar to determine intracellular CFU. Data are the mean (±SD) of three separate experiments. *Different superscripts* in different cell lines are indication of significance (*P* < *0.05*)

Figure 1 shows the invasion capacity of the different isolates in the three cell lines. Based on the invasion capacity into the human osteoblast SaOS-2 cells, two groups of three isolates each could be distinguished. The MDR F28 and drug susceptible KZN isolates grouped together with H37Rv, displaying significantly lower invasion capacity as compared to the second group which included the drug susceptible F11 and the MDR as well as XDR representative of the KZN strain (P ≤ 0.001).

In A549 alveolar epthelial cells, the XDR KZN and the MDR F28 isolates showed almost equal invasion levels (P > 0.05) and these were significantly higher than those of the susceptible KZN and the susceptible F11 isolates as well as H37Rv (P ≤ 0.001). The MDR KZN isolate featured in between with no significant difference with either of those two groups (P < 0.05). H37Rv invaded the mouse alveolar macrophage cell line MHS at levels reported by others (Liu et al. 2013; Sirakova et al. 2003). Like in alveolar epthelial cells, invasion capacity into MHS alveolar macrophages was highest for the XDR KZN and MDR F28 isolates and the difference with the other isolates was statistically significant (P ≤ 0.001). Drug susceptible F11 and both the drug susceptible KZN and MDR KZN isolates, showed similar invasion capacity as H37Rv. Between the two alveolar cell lines, invasion of MHS cells was higher for all isolates (P ≤ 0.008).

### Intra-cellular multiplication of *Mycobacterium tuberculosis*

**Fig. 2:**
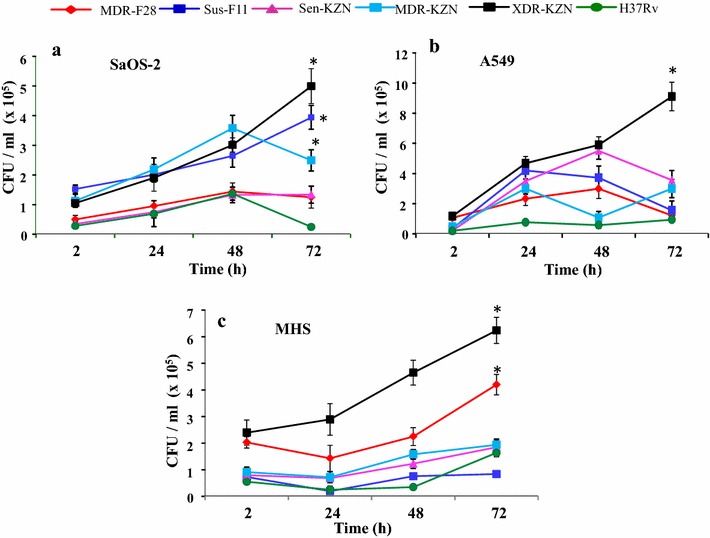
Intracellular growth of different *Mycobacterium tuberculosis* strains within **a** human osteoblasts (SaOS-2) cells, **b** human epithelial cells (A549) and **c** murine alveolar macrophages (MHS). After infection at MOI 9-12, cells were incubated with antibiotics to kill extracellular bacteria. Cells were further cultured in their specific media on 37 °C in 5 % CO_2_ atmosphere for different time hours to see the growth of different strains in different cell types. After specific time points cells were lysed and plated on 7H11 agar to determine intracellular CFU/ml. Data are the mean (±SD) of three separate experiments. (*) Indicates significant value (*P* < *0.05*)

Intracellular multiplication in bone cells and alveolar cells differed between isolates. In SaOS-2 cells the drug susceptible F11 and both the MDR and XDR KZN isolates showed the same multiplication pattern. The intracellular number of organisms increased over time at similar rates up to 48 h, with significantly higher number of organisms present at each time point as compared with the other isolates. The calculated growth rate was 0.62 × per 12 h. Between 48 and 72 h, the F11 and XDR KZN isolates kept multiplying with a growth rate of 0.75 × per 12 h and 0.83 × per 12 h respectively, but the MDR KZN growth rate became negative (−0.35 × per 12 h) in that time period. The drug susceptible KZN and MDR F28 isolates showed a multiplication rate of 0.81 × per 12 h between 2 and 48 h but growth of H37Rv became negative in the last 24 h (Fig. 2a).

In A549 cells, the intracellular number of organisms of the XDR KZN and drug susceptible KZN isolates increased at similar rate (0.69 × per 12 h) up to 48 h and after that time point XDR KZN isolates showed a progressive increase with a multiplication rate of 0.77 × per 12 h from the 48-h time point to 72 h of incubation. The growth rate of the drug susceptible KZN isolate became negative (−0.33 × per 12 h) in that time period. After 24 h, MDR F28 and drug susceptible F11 isolates exhibited a similar multiplication pattern with negative growth rate (−0.2 × per 12 h) from 48 to 72 h. Multiplication of the MDR KZN isolate stopped after 24 h and consequently over the next 48 h the number of organisms remained stable. Multiplication of H37Rv was significantly slower than any of the clinical isolates during the 72 h of incubation (Fig. 2b).

Intracellular multiplication in alveolar macrophages differed between isolates. In MHS cells the drug susceptible F11 and both the drug susceptible KZN and MDR KZN isolates showed the same multiplication pattern. After initial entry, the intracellular number of organisms of all three isolates displayed a slow growth rate (both KZN isolates) or numbers remained stable (F11) up to 24 h and then increased up to 72 h. The calculated growth rate during that period was 0.72 × per 12 h. The MDR F28 isolates showed a decrease in numbers during the first 24 h but then started to increase at a multiplication rate of 0.73 × per 12 h between 24 and 72 h. At each time point a significantly (P ≤ 0.001) higher number of organisms of the XDR KZN isolate was present when compared to the other isolates. The calculated growth rate was 0.54 × per 12 h between 24 and 72 h. H37Rv displayed a slow growth rate up to 48 h with acceleration in the last 24 h. This was comparable with F11 and the susceptible and MDR-KZN isolates but significantly (*P* < *0.05*) lower compared to XDR KZN or MDR F28 (Fig. 2c).

## Discussion

Extra-pulmonary tuberculosis occurs in any part of the body. The port of entry for *M. tuberculosis* is the lung. Spread through the body is thought to occur by transport inside migrating macrophages as well as through bacteraemia (Dannenberg and Rook 1994; Edwards and Kirkpatrick 1986). The site where the bacteria settle and later on develop extra-pulmonary tuberculosis was thought to be random. However, some recent studies reported associations between *M. tuberculosis* strains and site of extra-pulmonary disease (Adlakha et al. 2012; Be et al. 2012; Caws et al. 2008; Click et al. 2012; Garcia de Viedma 2003; Hernandez Pando et al. 2010; Lari et al. 2009). We postulated that the development of extra-pulmonary tuberculosis depends on the ability of the microbe to pass effectively through the alveolar epithelial lining and its affinity for cells other than those in pulmonary tissue. Since Pott’s disease is a frequently diagnosed form of extra-pulmonary tuberculosis, we chose human osteoblasts as the extra-pulmonary cell type. Hoshino et al. (2014) reported that *M. tuberculosis* strains can multiply in bone cells. However, they used osteoclasts in stead of osteoblasts. We chose osteoblasts based on the findings of Wright and Friedland (2002) who reported that in these cells the production of pro-inflammatory cytokines is upregulated when infected with *M. tuberculosis* or *S. aureus.* It is therefore likely that osteoblasts are essential in the development of Pott’s disease.

The MHS mouse macrophage cell line exposed to *M. tuberculosis* H37Rv served as control. We found that there are indeed major differences between the strains of *M. tuberculosis*. Based on former work in our laboratory with A549 cells in which we found differences between F15/LAM4/KZN isolates, we also included isolates of this strain with different resistance profiles (Ashiru et al. 2010).

Our results indicate that there are differences in invasion and intracellular multiplication between strains in alveolar epithelium and that for F15/LAM4/KZN invasion capacity increases with the level of resistance. Of the other two strains tested, the F28 strain showed similar invasion capacity as the XDR F15/LAM4/KZN. Whether this is related to the fact that the F28 isolate used had the MDR resistance profile needs further investigation. The observations with the osteoblast cell line differed in that now, the fully susceptible F11 isolate was the most invasive strain. Interestingly, while the susceptible F15/LAM4/KZN isolate’s invasion capacity in osteoblasts was low and comparable with H37Rv, the MDR and XDR isolates of this strain invaded at a much higher rate. Similarly, both resistant isolates of F15/LAM4/KZN showed a significantly higher multiplication rate in these cells. We conclude that in parallel to the development to MDR, a genetic event occurred that increased the affinity of the strain to bone tissue. This needs further exploration.

We explained the difference in the number of intracellular organisms between the isolates by differences in multiplication rate. We considered the possibility that the isolates with lower intracellular numbers were cytotoxic and therefore had fewer or less viable cells available. Although we did not test for cytotoxicity, this explanation seems unlikely since we did not observe any microscopic differences between the SaOS-2 cells exposed to the different isolates.

Passing of the mycobacteria through lung tissue into other parts of the body can be through transepithelial migration (Birkness et al. 1999) or through destruction of lung tissue (Danelishvili et al. 2003). Whether transepithelial migration occurs needs to be investigated. However, we do have evidence that cytoxic effect on A549 cells is highest in resistant isolates of F15/LAM4/KZN (Ashiru and Sturm 2015).

Most studies on interaction of *Mycobacterium tuberculosis* with alveolar cell types involve alveolar macrophages (Crouch et al. 2000; McCormack and Whitsett 2002). These large pleomorphic cells are found within lung alveoli as free cells or attached to the respiratory epithelium and are classically thought to be the first cell to respond to inhaled pathogens (Geiser 2002; Crouch et al. 2000; McCormack and Whitsett 2002). MHS cells are a useful model for the in vitro study of alveolar macrophages. Like in primary alveolar macrophages, *Mycobacterium tuberculosis* survives in MHS cells after infection (Melo and Stokes 2000). Our findings are in agreement with previous studies that *Mycobacterium tuberculosis* strains invade both A549 and MHS cells. However our results revealed that different strains showed distinct invasion capacity for different cell types and in general invasion capacity in MHS cells was higher compared to A549. This is similar with the findings of Mehta et al. (1996) who reported that in the epithelial cells bacterial uptake may be at a lower rate compared with macrophages (Mehta et al. 1996). The contribution of invasion of each of these alveolar cell types in the further development to extra-pulmonary tuberculosis needs additional investigations.

## Conclusion

It has been previously assumed that the site of extra-pulmonary tuberculosis (EPT) in individual patients was an event likely to be random, or at most influenced by host factors only. Recently, there is accumulating evidence that shows significant differences between strains of *Mycobacterium tuberculosis*. In this paper we postulated that the site of EPT was at least partly determined by bacterial characteristics.
